# Generation and Disruption of Circadian Rhythms in the Suprachiasmatic Nucleus: A Core-Shell Model

**DOI:** 10.1177/07487304221107834

**Published:** 2022-07-17

**Authors:** Alexander V. Goltsev, Edgar A. P. Wright, José F. F. Mendes, Sooyeon Yoon

**Affiliations:** Department of Physics & I3N, University of Aveiro, Aveiro, Portugal

**Keywords:** circadian rhythms, suprachiasmatic nucleus, dissociation, entrainment, Aschoff’s rule, Kuramoto model

## Abstract

We focus our research on how the core-shell organization controls behavior of the suprachiasmatic nucleus (SCN), how the core and shell are synchronized to the environment, what impact they have on the behavior of the SCN under different lighting conditions, and what mechanisms disrupt synchronization. To this end, we use a reduced Kuramoto model, with parameters inferred from experimental observations and calibrated for mice, and perform a detailed comparison between the model and experimental data under light-dark (LD), dark-dark (DD), and light-light (LL) conditions. The operating limits of free-running and entrained SCN activity under symmetric LD cycles are analyzed, with particular focus on the phenomena of anticipation and dissociation. Results reveal that the core-shell organization of the SCN enables anticipation of future events over circadian cycles. The model predicts the emergence of a second (dissociated) rhythm for large and small LD periods. Our results are in good qualitative and quantitative agreement with experimental observations of circadian dissociation. We further describe SCN activity under LL conditions and show that our model satisfies Aschoff’s first rule, according to which the endogenous free-running circadian period observed under complete darkness will shorten in diurnal animals and lengthen in nocturnal animals under constant light. Our results strongly suggest that the Kuramoto model captures essential features of synchronization and entrainment in the SCN. Moreover, our approach is easily extendible to an arbitrary number of groups, with dynamics described by explicit equations for the group phase and synchronization index. Viewed together, the reduced Kuramoto model presents itself as a useful tool for exploring open problems in the study of circadian rhythms, one that can account for evolving views of the circadian system’s organization, including peripheral clocks and inter-hemispheric interaction, and can be translated to other nocturnal and diurnal animals, including humans.

Circadian rhythms are periodic physiological and behavioral changes that take place in living organisms over 24-h cycles. Following decades of experimental and theoretical work, it is now understood that circadian rhythms are orchestrated by the brain’s suprachiasmatic nucleus (SCN) located in the hypothalamus (Evans and Gorman, 2016; Michel and Meijer, 2020; Silver, 2018). These endogenous rhythms are entrained to environmental cues such as light, and their disruption has a significant and sometimes detrimental impact on bodily function, including jet lag (disrupted sleep resulting from rapid travel across time zones), dissociation (emergence of a second rhythm in the SCN beyond the entrainment range, that is, for sufficiently large or small light-dark [LD] periods), and splitting (antiphase rhythms within the SCN) (Pittendrigh and Daan, 1976c; de la Iglesia et al., 2000; Silver, 2018; Michel et al., 2013). Despite considerable progress in our understanding of the SCN, the role of its organization in the generation of circadian rhythms remains an open problem (Hastings et al., 2018). At the population level, the current paradigm of SCN organization is based on 2 interconnected parts, the ventral (core) and dorsal (shell) regions of the SCN (Leak et al., 1999; Abrahamson and Moore, 2001; Evans and Gorman, 2016; Michel and Meijer, 2020; Silver, 2018; Hastings et al., 2018) where only the core is retinorecipient (Meijer et al., 1986). At the single cell level, self-sustained circadian rhythms are known to originate in feedback loops involving the transcription of clock genes (Ko and Takahashi, 2006), which persist in the absence of exogenous cues.

One approach to modeling circadian rhythms is based on molecular models of individual neuron activity (Leloup and Goldbeter, 2003; Vasalou and Henson, 2011; Hafner et al., 2012; Taylor et al., 2017). However, a single hemisphere of the SCN comprises approximately 10,000 neurons so that molecular models require a large number of equations and parameters to characterize both individual cells and the network organization of the SCN. This makes such models computationally harder and less amenable to analytical analysis. Moreover, experimental evidence and simplified theoretical models show that population-level activity in the SCN emerges from the dynamics of individual neurons (Schibler et al., 2015; Gu et al., 2016, 2018). Among simplified models of SCN dynamics, the Kuramoto model is a biologically plausible approach since clock cells in the SCN demonstrate self-sustained, nearly sinusoidal oscillations. It accounts for the heterogeneity of free-running periods observed across individual cellular oscillators (neurons) in the SCN (Liu et al., 1997; Pauls et al., 2014; Welsh et al., 2010; Taylor et al., 2017). The Kuramoto model has been used to explain the east-west asymmetry in jet lag recovery times (Lu et al., 2016), seasonal adaptation of the circadian clock (Gu et al., 2016; Meijer et al., 2010), and phase splitting within the SCN (Rohling and Meylahn, 2020). With few exceptions (Hannay et al., 2020), existing work either does not account for the core-shell organization of the SCN, which continues to guide considerable research into the functioning of the SCN (Evans et al., 2015; Evans and Gorman, 2016; Michel and Meijer, 2020; Silver, 2018), or explicitly models the dynamics of individual neurons.

Here, we mainly consider how the core-shell organization of the SCN controls SCN behavior, how the core and shell are synchronized to the environment, what impacts they have on the behavior of the SCN under different lighting conditions, and the mechanisms that disrupt synchronization. For this purpose, we use a reduced Kuramoto model, which explicitly describes the phase and synchronization index of the core and shell. The benefit of our model is that it uses only 9 biologically meaningful parameters, which can be inferred from experiments. Using this model, we study the operating limits of free-running and entrained SCN activity under symmetric LD, dark-dark (DD), and light-light (LL) cycles. In particular, we consider how the core-shell organization of the SCN enables anticipation of regular events and the emergence of a dissociated rhythm under different lighting conditions. Model parameters are inferred from experimental observations for mice and can be calibrated for other nocturnal and diurnal mammals. Our numerical and analytical results demonstrate good qualitative and quantitative agreement with experimental results.

## Methods

### The Reduced Kuramoto Model

According to anatomical data, neurons in the SCN are densely interconnected (Güldner, 1976; Moore and Bernstein, 1989; Varadarajan et al., 2018). Each SCN neuron forms between 300 and 1000 connections with other SCN neurons. Such dense connectivity means that many important features of SCN dynamics can be described by a mean-field model with all-to-all interaction (Dorogovtsev et al., 2008). The SCN in the left and right hemispheres comprises 2 main groups of clock cells, namely, the core and shell. As we have noted in the Introduction, since clock cells in the SCN demonstrate self-sustained, nearly sinusoidal oscillations, one can model them as weakly nonlinear oscillators with a stable limit cycle (Liu et al., 1997). The dynamics of this kind of oscillators is described by the Kuramoto model. Let us consider a generalized Kuramoto model where N heterogeneous phase oscillators are divided into M groups (or communities). Each oscillator i is characterized by a phase $\theta_{i}^{\left(m\right)}$ and has its own natural angular frequency $\omega_{i}^{\left(m\right)}$ , where m is the group index. Each group comprises $N_{m}$ oscillators, coupled with strength $K_{mm}$ within groups and strength $K_{nm}$ between groups. In a periodic external field with period T and angular frequency $\omega_{F}=2\pi /T$ , the local field phase $\omega_{F}t+\phi^{\left(m\right)}$ and strength $F^{\left(m\right)}$ can differ between groups. If interaction between the oscillators is absent, then the time evolution of individual Kuramoto oscillators is

(1) $$\begin{array}{l} \frac{d\theta_{i}^{\left(m\right)}}{dt}=\omega_{i}^{\left(m\right)}+F^{\left(m\right)}\sin(\omega_{F}t+\phi^{\left(m\right)}-\theta_{i}^{\left(m\right)}). \end{array}$$

In a system with interaction, the time evolution of the phase $\theta_{i}^{\left(m\right)}$ of each Kuramoto oscillator is

(2) $$\begin{array}{l} \frac{d\theta_{i}^{\left(m\right)}}{dt}=\omega_{i}^{\left(m\right)}+\frac{1}{N_{n}}\sum_{n}\sum_{j\in G_{n}}K_{nm}\sin(\theta_{j}^{\left(n\right)}-\theta_{i}^{\left(m\right)})\\ \;+F^{\left(m\right)}\sin(\omega_{F}t+\phi^{\left(m\right)}-\theta_{i}^{\left(m\right)}). \end{array}$$

The macroscopic state of each group is characterized by the synchronization index $\rho_{m}$ and group phase $\psi_{m}$ of the complex order parameter

(3) $$z_{m}=\rho_{m}e^{i\psi_{m}}\equiv \frac{1}{N_{m}}\sum_{j\in G_{m}}e^{i\theta_{j}^{\left(m\right)}}.$$

The amplitude (synchronization index) $\rho_{m}$ characterizes the extent of phase alignment between oscillators in group m and varies between 0 and 1 . When $\rho_{m}=0$ , oscillators within group m are in an asynchronous state, while $\rho_{m}=1$ corresponds to a completely synchronized state. The group phase $\psi_{m}$ is the average phase or direction of the oscillators.

**Figure 1.**
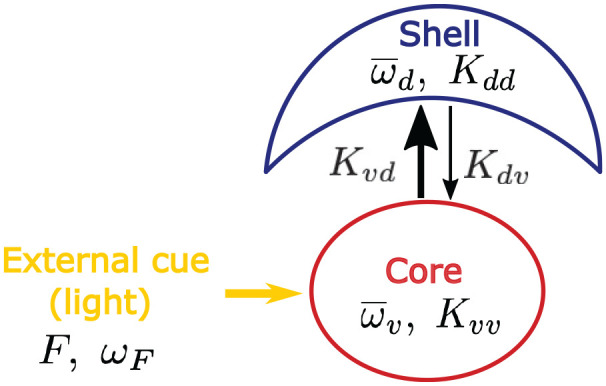
Schematic representation of the core-shell model consisting of 2 groups of oscillators, the core (ventral part υ ) and shell (dorsal part d ). Parameters F and $w_{F}$ are the strength and frequency of an external light cue acting exclusively on the core. The core (shell) is characterized by a mean free-running frequency ${\bar{w}}_{u(d)}$ , the statistical spread of free-running frequencies ${∆}_{u(d)}$ (Lorentzian half width at half maximum), and the intracoupling $K_{uu(dd)}$ between oscillators within the core (shell). Coupling between core and shell oscillators is determined by intercouplings $K_{ud}$ (core on shell) and $K_{du}$ (shell on core).

Here, we restrict our attention to a 2-group version of equation (2), with which we will model the core-shell organization and dynamics of the SCN. As shown elsewhere, equation (2) can be reduced to explicit equations for the phase and amplitude of each group (Ott and Antonsen, 2008; Yoon et al., 2021). Following this approach, the time dependence of the amplitude and phase in the core ( υ ) and shell ( d ) becomes

(4) $$\begin{array}{l} \frac{d\rho_{\upsilon }}{dt}=-\rho_{\upsilon }\Delta_{\upsilon }+\frac{1}{2}\rho_{\upsilon }K_{\upsilon \upsilon }(1-\rho_{\upsilon }^{2})\\ \;+\frac{1}{2}F(1-\rho_{\upsilon }^{2})\cos\psi_{\upsilon }\\ \;+\frac{1}{2}\rho_{d}K_{d\upsilon }(1-\rho_{\upsilon }^{2})\cos(\psi_{d}-\psi_{\upsilon }), \end{array}$$

(5) $$\begin{array}{l} \frac{d\psi_{\upsilon }}{dt}=-\Omega_{\upsilon }-\frac{1}{2}F\frac{\left(1+\rho_{\upsilon }^{2}\right)}{\rho_{\upsilon }}\sin\psi_{\upsilon }\\ \;+\frac{1}{2}\rho_{d}K_{d\upsilon }\frac{\left(1+\rho_{v}^{2}\right)}{\rho_{v}}\sin(\psi_{d}-\psi_{\upsilon }), \end{array}$$

(6) $$\begin{array}{l} \frac{d\rho_{d}}{dt}=-\rho_{d}\Delta_{d}+\frac{1}{2}\rho_{d}K_{dd}(1-\rho_{d}^{2})\\ \;+\frac{1}{2}\rho_{\upsilon }K_{\upsilon d}(1-\rho_{d}^{2})\cos(\psi_{\upsilon }-\psi_{d}), \end{array}$$

(7) $$\frac{d\psi_{d}}{dt}=-\Omega_{d}+\frac{1}{2}\rho_{\upsilon }K_{\upsilon d}\frac{\left(1+\rho_{d}^{2}\right)}{\rho_{d}}\sin(\psi_{\upsilon }-\psi_{d}),$$

in the frame rotating at the external field frequency $\omega_{F}$ . The field phase is set to 0 since it provides a reference time for the external cue. Equations (4)-(7) describe the core-shell model schematically depicted in Figure 1, where the parameters characterizing the heterogeneity of dynamical properties among SCN oscillators are reduced to a set of parameters characterizing the mean properties of the core and shell. These include the characteristic mean ${\bar{\omega }}_{\upsilon }$ ( $c_{d}$ ) and half width at half maximum ${∆}_{\upsilon }$ ( ${∆}_{d}$ ) of the natural frequency distribution in the core (shell). At a given field frequency, the mean natural frequency determines the detuning $\Omega_{\upsilon }=\omega_{F}-{\bar{\omega }}_{\upsilon }$ in the core and $\Omega_{d}=\omega_{F}-{\bar{\omega }}_{d}$ in the shell. Note that a periodic external field is taken explicitly into account by equations (4)-(7), which allows to study nonlinear impacts of the external field on the SCN. At $K_{\upsilon d}=K_{d\upsilon }=0$ , these equations are reduced to the explicit equations for the forced Kuramoto model derived in Childs and Strogatz (2008).

In exchange for the simplification of equation (2) into equations (4)-(7), the natural frequencies or free-running periods of SCN oscillators are assumed to follow a Cauchy-Lorentz distribution. Despite the restriction, the Cauchy-Lorentz distribution is unimodal and symmetric, like the Gaussian distribution, which has been used to model the distribution of free-running periods in the core and shell (Taylor et al., 2017). From the physical point of view, the Kuramoto model demonstrates qualitatively similar behavior for both the Cauchy-Lorentz and Gaussian distributions of natural frequencies. The main difference is in their analytical properties. The Cauchy-Lorentz distribution has residuals in the complex plane. This fact simplifies analytical calculations and allows an explicit reduction of microscopic equation (2) to a set of equations (4)-(7) that describe the evolution of the macroscopic characteristics of the Kuramoto model.

Note that stationary values of the synchronization indices $\rho_{\upsilon }$ and $\rho_{d}$ can be found analytically by solving equations (4)-(7) at zero time derivatives on the left hand side. Unfortunately, the strong non-linearity of these equations makes it difficult to find a simple analytical expression, while numerical solutions can be readily computed.

Recently, a model similar to our model in Figure 1 was considered by Hannay et al. (2020). Using the Ott-Antonsen ansatz, Hannay et al. (2020) derived equations similar to equations (4)-(7) to describe the evolution of the core and shell. While Hannay et al. (2020) focused on seasonal day length, after-effects of light entrainment, and seasonal variations in light sensitivity in the mammalian circadian clock, in this article, we mainly focused on (1) dynamical behavior of the core and shell under LD, DD, and LL conditions, including anticipation in nocturnal animals; (2) dependence of the entrainment range on the properties of core and shell neurons; (3) the mechanisms of circadian rhythm dissociation with increasing (or decreasing) LD cycle period above (or below) a critical threshold; and (4) dissociation under LL conditions in nocturnal and diurnal animals. Another important difference between the work of Hannay et al. (2020) and our own lies in how the external cue (i.e., light) is taken into account. In our approach, a periodic external optic cue is explicitly taken into account by equations (4)-(7). This enables to study nonlinear effects in the impact of the cue on the SCN. Hannay et al. considered only a collective phase response on an external cue, assuming that a light perturbation shifts mean-field phases.

In summary, our approach is based on the observation that clock cells in the SCN are self-sustained, nearly sinusoidal oscillators with a stable limit cycle (Liu et al., 1997). Individual dynamics of this kind of oscillators can be approximated by a simple differential equation (1). Accounting for interactions between oscillators, we obtain equation (2), which provides a complete description at the microscopic (cell) level. Finally, using explicit mathematical methods, we reduce the microscopic equation to equations (4)-(7), which describe the macroscopic dynamics of the core and shell. The assumption, that the SCN cells are self-sustained and nearly sinusoidal oscillators, may not hold true in general. Account of processes that disturb these conditions and lead to non-sinusoidal individual dynamics is an open problem. In this regard, one possible line of research, beyond the scope of the present work, is to compare models with distinct clock cell dynamics.

### Selecting Model Parameters

This section discusses the choice of parameters used in the numerical study of equations (4)-(7). For convenience, numerical calculations were performed using dimensionless parameters, with reference to frequency unit ${∆}_{\upsilon }$ . This is equivalent to dividing both sides of equations (4)-(7) by ${∆}_{\upsilon }$ , rescaling all parameters $K_{nm}/\Delta_{\upsilon }\to K_{nm}$ and time $t\Delta_{\upsilon }\to t$ . Dimensionless parameters are summarized in Table 1, along with their dimensional equivalents, and reference values are found in the literature.

**Table 1.** List of parameters (parameters) and a summary of the corresponding values reported by Taylor et al. (2017) (reference), the core-shell model (model) of equations (4)-(7).

Parameters	Reference	Model
$\tau_{\upsilon }$	25.1 h	25.1 h
$\tau_{d}$	23.9 h	23.3 h
$\sigma_{\upsilon }$	1.3 h	1.3 h
$\sigma_{d}$	1.9 h	1.9 h
$K_{\upsilon \upsilon }$	—	5.6
$K_{dd}$	—	4.0
$K_{\upsilon d}$	—	1.1
$K_{d\upsilon }$	—	0.5
F	—	1.5
${\bar{\omega }}_{\upsilon }$	—	19.3
${\bar{\omega }}_{d}$	—	20.8
${∆}_{\upsilon }$	—	1.0
${∆}_{d}$	—	1.7

Time-dimensional parameters include $\sigma_{\upsilon (d)}$ , the standard deviation of free-running periods reported by Taylor et al. (2017); $\tau_{\upsilon (d)}$ , the mean free-running period of the core (shell). Dimensionless parameters are obtained by multiplying time-dimensional parameters and dividing frequency-dimensional parameters by the frequency unit $\Delta_{\upsilon }=\pi \sigma_{\upsilon }/\tau_{\upsilon }^{2}$ . Frequency-dimensionless parameters include $K_{\upsilon \upsilon (dd)}$ , the intracoupling in the core (shell); $K_{\upsilon d}$ and $K_{d\upsilon }$ , the core-shell intercouplings; F , the strength of the light cue acting exclusively on the core. The dimensionless equivalents of the model parameters: ${\bar{\omega }}_{\upsilon (d)}=2\pi /\tau_{\upsilon (d)}$ , the mean free-running frequency of core (shell) oscillators; and ${∆}_{\upsilon (d)}$ , the Lorentzian spread (half width at half maximum) of free-running frequencies in the core (shell).

The choice of ${\bar{\omega }}_{\upsilon (d)}$ and ${∆}_{\upsilon (d)}$ is based on a study of mice exposed to symmetric 12-h LD cycles by Taylor et al. (2017). In the present core-shell model, 12-h LD cycles correspond to sequential half cycles of the field with period T=24h . Based on experimental observations, Taylor et al. (2017) fitted the free-running periods of the core ( υ ) and shell ( d ) to Gaussian distributions with mean $\tau_{\upsilon }=25.1\;h$ and $\tau_{d}=23.9\;h$ , respectively, and standard deviation $\sigma_{\upsilon }=1.3\;h$ and $\sigma_{d}=1.9\;h$ . The mean free-running periods can be directly converted to mean free-running frequencies ${\bar{\omega }}_{\upsilon (d)}=2\pi /\tau_{\upsilon (d)}$ . From the definition of standard deviation, and given $\tau_{\upsilon (d)}\gg \sigma_{\upsilon (d)}$ , the standard deviation of the free-running frequencies is approximated as $SD_{\upsilon (d)}\approx 2\pi \sigma_{\upsilon (d)}/\tau_{\upsilon (d)}^{2}$ . Although the present core-shell model is based on a Lorentzian distribution of free-running frequencies with half width at half maximum ${∆}_{\upsilon (d)}$ , Gaussian and Lorentzian distributions both describe a system where most neurons have a free-running period approximately equal to the system average, and the number of neurons with larger or smaller than average free-running frequencies is symmetric and monotonically decreasing. In both cases, ${∆}_{\upsilon (d)}$ and $SD_{\upsilon (d)}$ provide a measure of the statistical spread of free-running frequencies about the mean value, so we simply take ${∆}_{\upsilon (d)}=SD_{\upsilon (d)}$ .

The coupling mechanisms between SCN neurons are an ongoing topic of research (see, for example, Tokuda et al., 2018; Hastings et al., 2018; and references therein). Although direct measurements of intra- and intercouplings $K_{\upsilon \upsilon }$ , $K_{dd}$ , $K_{\upsilon d}$ , and $K_{d\upsilon }$ are not available in the literature, these may be inferred from experimental observations by treating coupling as a proxy for neuron connectivity/communication. When inferring parameters, we may also restrict ourselves to values for which the system can sense and adjust to small changes in the external cue. At one extreme, excessively large coupling ( $K_{\upsilon \upsilon },K_{dd},K_{\upsilon d},K_{d\upsilon }\gg 1$ ) makes the system insensitive to the external cue. At the other extreme, an external cue of excessively large strength ( F≫1 ) makes it impossible for the system to adjust its state through coupling changes, such as may be induced by neuronal plasticity (Rohr et al., 2019). Thus, realistic couplings and cue strength should be selected from an intermediate range where SCN plasticity is possible.

From a functional point of view, studies of SCN slices in the mouse by Yamaguchi et al. (2003) and rat by Noguchi et al. (2004) have shown that the ventral SCN remains synchronized when isolated from the dorsal SCN. These observations suggest that coupling within the isolated ventral SCN (core) is large enough to support synchronization. By comparison, the isolated shell was only found to remain synchronized in the rat (Noguchi et al., 2004). From a structural point of view, dendritic arbors within the shell are sparse, whereas those within the core are more extensive and larger (Moore, 1996). This suggests that there are more synaptic contacts between neurons in the core than in the shell. Viewed together, functional and structural observations suggest that intra-core coupling $K_{\upsilon \upsilon }$ is larger than the critical value 2 ${∆}_{\upsilon }$ required to synchronize the isolated core and larger than the intra-shell coupling $K_{dd}$ . For simplicity, the intracouplings are estimated for the isolated core and shell under constant darkness. Although the intracoupling may vary in response to photic input if the core and shell are coupled, consequent changes in the state of the system may be captured by other model parameters, as discussed in the next paragraph. In the isolated core (shell) under constant darkness ( $K_{\upsilon d}=K_{d\upsilon }=0$ and F=0 ), intracoupling increases with the fraction of synchronized oscillators $\rho_{\upsilon (d)}$ as follows:

(8) $$K_{\upsilon \upsilon (dd)}=\frac{2\Delta_{\upsilon (d)}}{1-\rho_{\upsilon (d)}^{2}}\;\mathrm{or}\;\rho_{\upsilon (d)}=\sqrt{1-\frac{2\Delta_{\upsilon (d)}}{K_{\upsilon \upsilon (dd)}}},$$

which can be seen from equations (4) and (6). For positive intracoupling, it follows that $\rho_{\upsilon }>\rho_{d}$ whenever $K_{\upsilon \upsilon }/K_{dd}>$ ∆_v_/∆_d_, which is the case since $K_{\upsilon \upsilon }>K_{dd}$ and ∆_d_ > ∆_v_. In the absence of suitable experimental data with which to infer $\rho_{\upsilon (d)}$ , we take $\rho_{\upsilon }=2\rho_{d}$ , for example, and refer to computational models for a reasonable upper bound on $\rho_{\upsilon }$ . For instance, the theoretical model used by Taylor et al. (2017) results in an average synchronization index of 0.9 in the entrained SCN. Considering a slightly smaller value of $\rho_{\upsilon }=0.8$ in the isolated core, the corresponding shell value is $\rho_{d}=0.4$ . In dimensionless units, equation (8) then yields $K_{\upsilon \upsilon }=5.6$ and $K_{dd}=4.0$ . The critical value of the core (shell) intracoupling $K_{c}^{\upsilon \upsilon }=2$ ∆_v_ ( $K_{c}^{dd}=2$ ∆_d_) at which synchronization emerges follows directly from equation (8) in the limit where $\rho_{\upsilon (d)}=0$ . Thus, the chosen intracoupling is considerably closer to the critical value $K_{c}^{dd}=3.4$ in the shell, where $(K_{dd}-K_{c}^{dd})/K_{c}^{dd}=0.18$ , than to the critical value $K_{c}^{\upsilon \upsilon }=2.0$ in the core, where $(K_{\upsilon \upsilon }-K_{c}^{\upsilon \upsilon })/K_{c}^{\upsilon \upsilon }=1.8$ . “Near-critical” coupling in the shell is compatible with the idea that variability between species and/or individuals can result in coupling changes around the critical value and may explain why the isolated shell has been observed to synchronize in rats (Noguchi et al., 2004) but not in mice (Yamaguchi et al., 2003).

Regarding the intercouplings $K_{\upsilon d}$ and $K_{d\upsilon }$ , experimental evidence shows that there are far more contacts made by neurons expressing vasoactive intestinal polypeptide (VIP) in the core onto neurons expressing arginine vasopressin (AVP) in the shell than the converse (Varadarajan et al., 2018). In addition, functional studies of resynchronization dynamics in the SCN have revealed that the core entrains the shell (Taylor et al., 2017). Viewed together, such findings support the hypothesis that signaling from the core to the shell is stronger than the reverse ( $K_{\upsilon d}>K_{d\upsilon }$ ). However, different contributions to coupling by different neurotransmitters and neuropeptides may affect the sign of the coupling, which impacts entrainment boundaries, the emergence of unstable states, and splitting of activity rhythms into 2 synchronized bouts cycling in antiphase (Daan and Berde, 1978; Oda and Friesen, 2002; Evans et al., 2005; Evans and Gorman, 2016). For instance, the neurotransmitter gamma-aminobutyric acid (GABA) has been found to both inhibit and promote synchrony in the SCN network depending on the state of the SCN (Evans et al., 2013). In an antiphase configuration ( $\psi_{d}=\pi +\psi_{\upsilon }$ ) following prolonged light exposure, GABA works with the neuropeptide VIP to promote synchrony. In the steady-state induced by symmetric LD conditions, GABA counters the synchronizing effect of the neuropeptide VIP. Since experimental observations demonstrate that the core entrains the shell under symmetric LD conditions (Taylor et al., 2017), the inhibiting contribution of GABA signaling is assumed smaller than the synchronizing effect of VIP ( $K_{\upsilon d}>0$ ). Reasonable bounds on the intercouplings can also be established through analysis of equations (4)-(7) under symmetric LD conditions, as discussed in the next section, and constant darkness, as discussed in the section on DD conditions. In particular, equation (11) shows that $K_{\upsilon d}>{\bar{\omega }}_{d}-\omega_{F}=0.15$ , and equation (24) shows that $K_{\upsilon d}+K_{d\upsilon }>{\bar{\omega }}_{d}-{\bar{\omega }}_{\upsilon }=0.97$ . Starting from these bounds and intracouplings $K_{\upsilon \upsilon }=5.6$ and $K_{dd}=4.0$ (chosen in the previous paragraph), parameters $K_{\upsilon d}$ , $K_{d\upsilon }$ , F , and $\tau_{d}$ were adjusted to ensure agreement with experimental observations of the difference in peak activity time between the core and the shell $t_{\upsilon }^{\left(p\right)}-t_{d}^{\left(p\right)}$ . As shown further below in equation (12), the difference between the peak activity time of the core $t_{\upsilon }^{\left(p\right)}$ and the shell $t_{d}^{\left(p\right)}$ is directly related to the phase difference $\psi_{d}-\psi_{\upsilon }$ that characterizes the state of the entrained SCN network. The difference $t_{\upsilon }^{\left(p\right)}-t_{d}^{\left(p\right)}$ has been experimentally measured at 2.4±0.9h in the entrained SCN under symmetric LD conditions, based on peak expression of the Per2 gene (Taylor et al., 2017). Parameters $\tau_{d}$ , $K_{\upsilon d}$ , $K_{d\upsilon }$ , and F were adjusted by inspection, until $t_{\upsilon }^{\left(p\right)}-t_{d}^{\left(p\right)}=2.3\;h$ in the entrained state ( T=24h ), for $\tau_{d}=23.3\;h$ , $K_{\upsilon d}=1.1$ , $K_{d\upsilon }=0.5$ , and F=1.5 (dimensionless units).

Thus, the advantage of our approach based on the Kuramoto model is that it reduces the large number of parameters that characterize tens of thousands of neurons and their couplings within the SCN to only 9 parameters, which capture the mean characteristics of core and shell neurons (see Table 1): the free-running periods $\tau_{\upsilon }$ and $\tau_{d}$ of the core and shell; the standard deviation of the free-running periods, $\sigma_{\upsilon }$ and $\sigma_{d}$ ; the intracouplings $K_{\upsilon \upsilon }$ and $K_{dd}$ within the core and shell; the intercouplings $K_{\upsilon d}$ and $K_{d\upsilon }$ between the core and shell; and the strength of the optic cue F . These parameters were obtained from an analysis of experimental data (Taylor et al., 2017). The remaining 4 parameters in Table 1 ( $\omega_{\upsilon }$ , $\omega_{d}$ , ${∆}_{\upsilon }$ , ${∆}_{d}$ ) are dimensionless equivalents of the model parameters. As we will show in the next sections, these 9 parameters describe the dynamical properties of the SCN under 3 lighting conditions (LD, DD, and LL), anticipation in the shell, and circadian rhythm dissociation in response both to varying LD period and to varying light intensity under LL conditions, in qualitative and quantitative agreement with experimental observations for diurnal and nocturnal mammals.

## Results

### Core-Shell Phase Difference and Anticipation In The Entrained State

The SCN can trigger behavior in anticipation of regular events. Examples include water-seeking in advance of sleep time and food-seeking in advance of meal times (Silver, 2018; Gizowski et al., 2016). From an experimental point of view, existing evidence supports a model of the SCN where the core receives a photic cue and the shell is responsible for transmitting phase information to the wider circadian system. For example, anatomical evidence shows that vasopressin (VP) neurons in the shell provide output to downstream tissues (see Gizowski et al., 2016, and references therein), and functional evidence shows that the shell entrains the phase of cellular oscillators in downstream tissues (Evans et al., 2015).

This section investigates how the shell of the entrained SCN can provide a reference phase for anticipation. To this end, an analytical and numerical analysis of equations (4)-(7) is presented, using the parameters in Table 1. Entrainment corresponds to a stationary solution of equations (4)-(7), which describes entrainment in a frame rotating at the frequency of the external cue $\omega_{F}=2\pi /T$ . In an entrained system, the phase difference $\Delta \psi \equiv \psi_{d}-\psi_{\upsilon }$ between shell and core groups of oscillators is readily obtained from equation (7):

(9) $$\sin(\psi_{d}-\psi_{\upsilon })=\frac{2\rho_{d}({\bar{\omega }}_{d}-\omega_{F})}{\rho_{\upsilon }(1+\rho_{d}^{2})K_{\upsilon d}}.$$

When the synchronization index of the entrained SCN is large ( $\rho_{\upsilon }\approx \rho_{d}\approx 1$ ), or more generally when $2\rho_{d}\approx \rho_{\upsilon }(1+\rho_{d}^{2})$ , it follows that

(10) $$\sin(\psi_{d}-\psi_{\upsilon })\approx \frac{{\bar{\omega }}_{d}-\omega_{F}}{K_{\upsilon d}}.$$

Under these conditions, positive $K_{\upsilon d}$ must be larger than the difference between the mean natural frequency of the shell ${\bar{\omega }}_{d}$ and the frequency of the external cue $\omega_{F}$ :

(11) $$K_{\upsilon d}>{\bar{\omega }}_{d}-\omega_{F},$$

since $|\sin(\psi_{d}-\psi_{\upsilon })|\leq 1$ . Direct inspection of equation (9) also shows that the sign of the shell-core phase difference is determined by the sign of the shell’s detuning parameter $\Omega_{d}=\omega_{F}-{\bar{\omega }}_{d}$ and of the intercoupling $K_{\upsilon d}$ . Since the mean free-running period of the shell $\tau_{d}=23.9\;h$ is smaller than the period of the external cue T=24h , it follows that $\Omega_{d}=2\pi /T-2\pi /\tau_{d}<0$ . In this case, the phase difference $\Delta \psi =\psi_{d}-\psi_{\upsilon }$ is positive since $K_{\upsilon d}$ is also positive ( $K_{\upsilon d}=1.1$ ). The extent to which $\psi_{d}$ leads $\psi_{\upsilon }$ is determined by the period of LD cycles $T=\pi /\omega_{F}$ . As hinted by equation (9), the phase difference −π/2≤Δψ≤π/2 tends to decrease as T decreases below 24 h, toward the shell’s free-running period $\tau_{d}=23.3\;h$ , and tends to increase as T increases above 24h . In general, the entrained phase difference is also dependent on how the synchronization indices $\rho_{\upsilon }$ and $\rho_{d}$ vary with T .

In the laboratory (non-rotating) frame, the real part of core (shell) order parameter $z_{\upsilon (d)}$ in equation (3) oscillates with frequency $\omega_{F}=2\pi /T$ . Symbolically, $\mathrm{Re(}z_{\upsilon (d)})=\rho_{\upsilon (d)}\cos(\omega_{F}t+\psi_{\upsilon (d)})$ . This time-dependent observable acts as a proxy for SCN activity, encoding synchronization index and phase at a given cue frequency or period. Note that the peak in core activity is locked to the peak in the external cue because the photic cue in our core-shell model acts exclusively on the core.

The observable $\mathrm{Re(}z_{\upsilon (d)})$ is maximum in the core (shell) at time $t_{\upsilon (d)}^{\left(p\right)}=2\pi n/\omega_{F}-\psi_{\upsilon (d)}/\omega_{F}$ , where n is an integer number. From here, it follows that the difference in peak activity time between the shell and the core is

(12) $$t_{\upsilon }^{\left(p\right)}-t_{d}^{\left(p\right)}=\frac{T(\psi_{d}-\psi_{\upsilon })}{2\pi }.$$

Thus, the peak time difference $t_{\upsilon }^{\left(p\right)}-t_{d}^{\left(p\right)}$ is determined by the phase difference $\psi_{d}-\psi_{\upsilon }$ . Using equation (10), we find how far ahead in time is the shell activity with respect to the core activity in the dependence on the frequency of the endogenous cycles in the shell, $\omega_{d}$ , and the coupling $K_{\upsilon d}$ :

(13) $$t_{d}^{\left(p\right)}=t_{\upsilon }^{\left(p\right)}-\frac{T}{2\pi }\arcsin\left(\frac{{\bar{\omega }}_{d}-\omega_{F}}{K_{\upsilon d}}\right).$$

**Figure 2.**
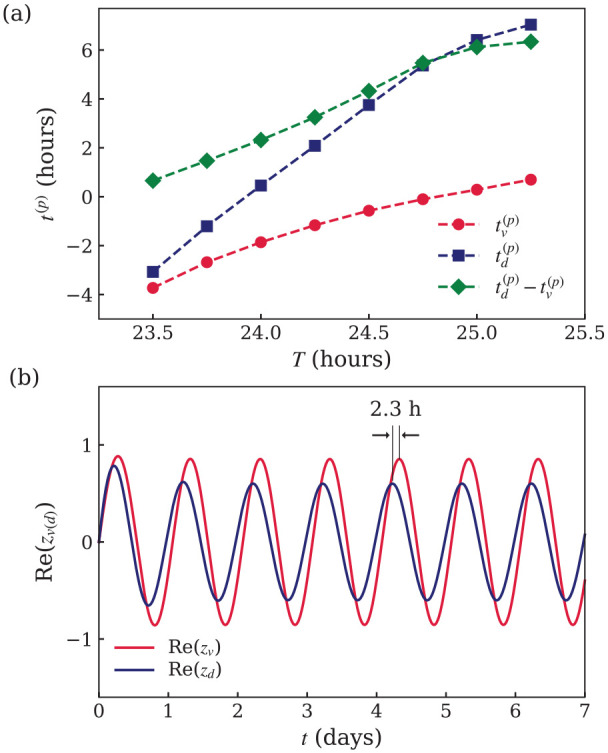
Results of numerical simulations of equations (4)-(7) with the parameters in Table 1. (a) Times $t_{\upsilon }^{\left(p\right)}$ (red circles) and $t_{d}^{\left(p\right)}$ (navy squares) at which the real part of complex order parameters $z_{\upsilon }$ and $z_{d}$ peak in the core and shell, respectively, and the core-shell difference between peak times $t_{\upsilon }^{\left(p\right)}-t_{d}^{\left(p\right)}$ (green diamonds) in the entrained state, as a function of the external light-dark period T . (b) Time dependence of the real part of the order parameter in the core ( $\mathrm{Re(}z_{\upsilon })$ , red line) and shell ( $\mathrm{Re(}z_{d})$ , navy line) for light-dark cycles with period T=24h . The peak time in the shell precedes the peak time in the core by 2.3h .

As discussed in the preceding paragraph, $\psi_{d}$ leads $\psi_{\upsilon }$ for positive $K_{\upsilon d}$ , since $\tau_{d}<T$ and therefore ${\bar{\omega }}_{d}-\omega_{F}>0$ . From equation (12), it then follows that with increasing time, the core must peak after the shell, that is, $t_{\upsilon }^{\left(p\right)}>t_{d}^{\left(p\right)}$ (see Figure 2b). At that time, the peak of the core activity is locked to the peak of the external cue. This result agrees with the experimental observation that peak Per2 expression in the shell precedes peak Per2 expression in the core by 2.4±0.9h (Taylor et al., 2017).

Viewed together, the results presented in this section show that the core-shell organization of the SCN enables the shell to anticipate events by advancing its phase relative to the phase of the core and the external cue while entrained to a symmetric LD cycle of period T . This lead is determined by the core-shell phase difference. For example, the numerical solution of equations (4)-(7) with the parameters in Table 1 shows that, for T=24 h, the lag $t_{\upsilon }^{\left(p\right)}-t_{d}^{\left(p\right)}=2.3\;h$ corresponds to the phase difference $\psi_{d}-\psi_{\upsilon }=0.607\;\mathrm{rad}\approx \mathrm{35}\;\mathrm{degrees}$ . Moreover, the shell can also increase its lead over the core in response to increasing T , as shown in Figure 2a.

To outline the importance of the anticipation, which is formed by the shell, we want to note that the SCN establishes phase coherence between self-sustained and cell-autonomous oscillators in peripheral organs, for example, liver, muscle, pancreas, heart, adipose tissue, and other areas of the brain (e.g., pineal gland which produces a hormone [melatonin] and modulates sleep-wake cycles). It is important to note that the organs receive signals mainly from the shell. This gives them the possibility to be prepared in advance for a change of activity. These organs are also influenced by other cues such as feeding/fasting cycles.

### Entrainment Range and Dissociation Under LD Conditions

Experimental investigations have revealed that animals can only entrain to a limited range of symmetric LD periods, known as the entrainment range: LLE<T<ULE , where LLE and ULE are the lower and upper limit of entrainment, respectively (Pittendrigh and Daan, 1976a; Campuzano et al., 1998; Usui et al., 2000; de la Iglesia et al., 2004; Goldbeter and Leloup, 2021). In nocturnal rodents, the entrainment range spans from LLE∼∼22h up to ULE∼∼28.5h , varying between species and individuals (Pittendrigh and Daan, 1976a; Campuzano et al., 1998; Usui et al., 2000). Beyond the entrainment range, it has been observed that animal behavior and SCN activity follow an additional rhythm, which differs from that of the external light cue. This phenomenon is known as dissociation (Campuzano et al., 1998; Usui et al., 2000; de la Iglesia et al., 2004; Schwartz et al., 2009).

**Figure 3.**
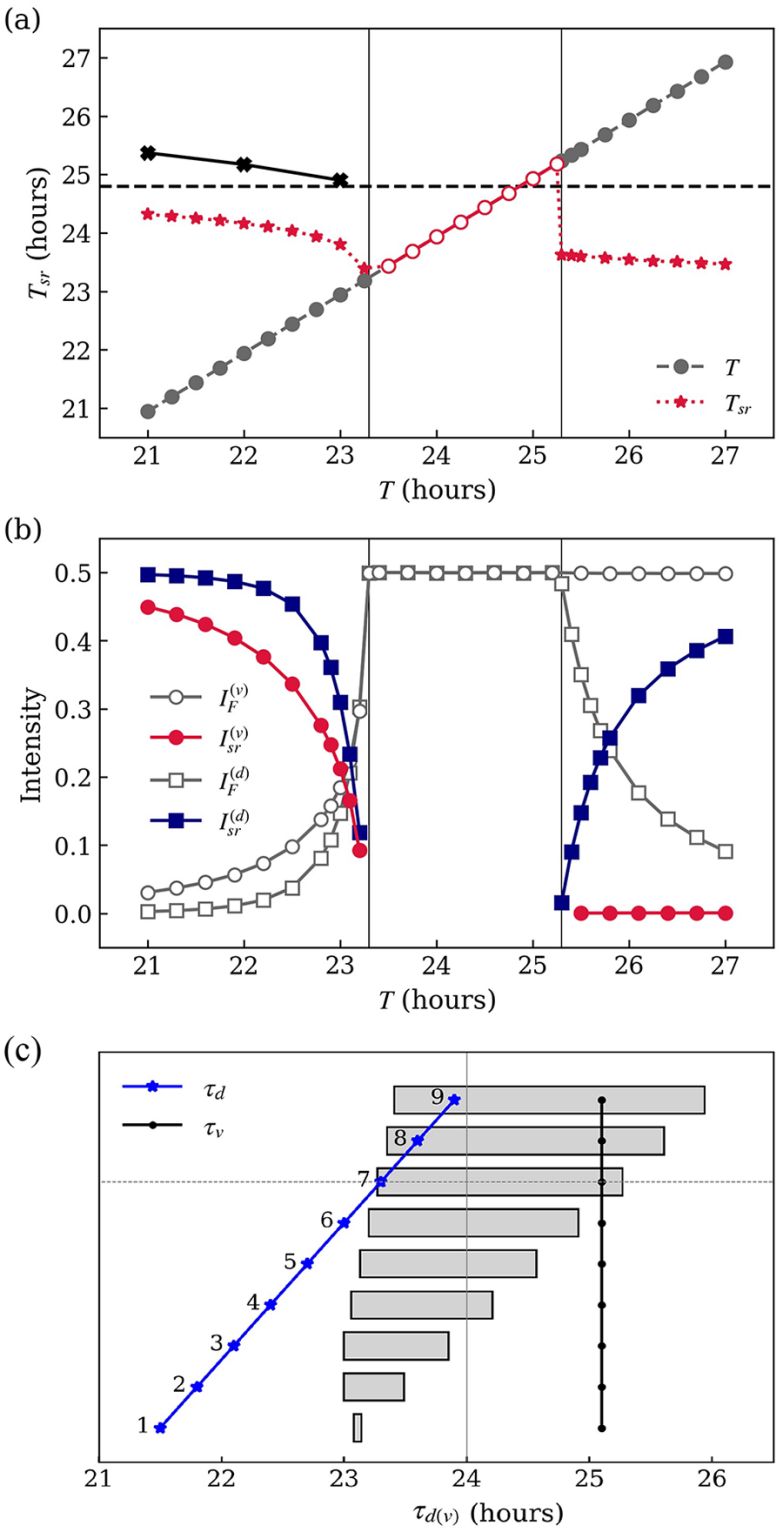
Results of numerical simulations of equations (4)-(7) with the parameters in Table 1. (a) Period $T_{sr}$ of independent SCN activity components under external light-dark cycles of varying period T (in hours). The component at the field period T is indicated in red open circles within the entrainment range and in gray circles outside the entrainment range. Red stars indicate the period of SCN activity at a second (dissociated) rhythm $T_{sr}$ . Black crosses are experimental data reported by Campuzano et al. (1998). The dashed horizontal line is the period of SCN oscillations $\tau_{DD}=24.84\;h$ under dark-dark conditions (cue strength F=0 ), calculated from the mean value of equations (5) and (7) in the synchronized state. (b) Intensity of light-dark ( F , hollow markers) and second rhythm ( sr , solid makers) components of SCN activity, in the core ( υ , circles) and shell ( d , squares). Vertical lines indicate the lower and upper bounds of the entrainment range, LLE=23.26h and ULE=25.28h , respectively. For comparison, the mean free-running periods of the shell and the core are $\tau_{d}=23.3\;h$ and $\tau_{\upsilon }=25.1\;h$ , respectively. (c) Dependence of the entrainment range (gray horizontal bars) on the difference between the free-running periods of core and shell, $\tau_{\upsilon }-\tau_{d}$ . Here, $\tau_{\upsilon }$ is fixed as $\tau_{\upsilon }=25.1\;h$ (black dots) and $\tau_{d}$ (blue dots) varies. Abbreviation: LLE = lower limit of entrainment;SCN = suprachiasmatic nucleus; ULE = upper limit of entrainment.

This section studies dissociation from the point of view of an observer in the laboratory frame. To this end, it is assumed that the observer measures the observable $\mathrm{Re(}z_{\upsilon (d)})=\rho_{\upsilon (d)}\cos(\omega_{F}t+\psi_{\upsilon (d)})$ or the corresponding distribution of frequency components $S_{\upsilon (d)}(\omega )$ (spectral density), as a proxy for SCN activity under varying LD period T . Here, the core (shell) synchronization index $\rho_{\upsilon (d)}$ and phase $\psi_{\upsilon (d)}$ are determined by numerical solution of equations (4)-(7), using the parameters in Table 1. The main results concerning independent components of the spectral density $S_{\upsilon (d)}(\omega )$ are presented in Figure 3.

**Figure 4.**
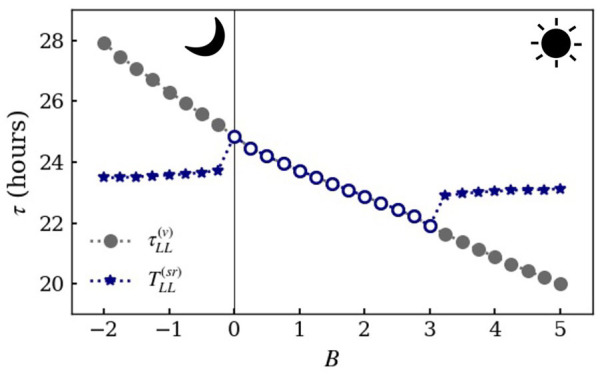
Period τ of independent activity components in the core as a function of parameter B under constant light conditions (from numerical simulations of equations (4)-(7) with the parameters in Table 1). The period $\tau_{LL}$ of the free-running component is indicated by circles, and the period $T_{LL}^{\left(sr\right)}$ of a second (dissociated) component is indicated by navy stars. Negative B corresponds to the nocturnal mouse (see Table 1 for parameters) and positive B to a fictitious diurnal counterpart. The second rhythm appears at $B_{c}≃-0.24$ in the nocturnal case and $B_{c}≃3.23$ in the diurnal case.

First, Figure 3a shows that a second rhythm ( sr ) with period $T_{sr}$ emerges outside the entrainment range. Above the upper bound ( ULE=25.28h ), $T_{sr}$ is smaller than T and decreases with increasing T . Below the lower bound ( LLE=23.26h ), $T_{sr}$ is larger than T and increases with decreasing T . These results are in qualitative agreement with experimental observations in rats (Campuzano et al., 1998; Usui et al., 2000). For instance, Usui et al. (2000) studied recurring drinking behavior in rats exposed to LD cycles with period T and report the emergence of a second recurring peak in drinking activity with period $T_{sr}=25\;h$ at T=ULE=28.5h (see Figure 3 by Usui et al., 2000), and $T_{sr}=23.5\;h$ at T=LLE=23h (see Figure 4 by Usui et al., 2000). The authors also report that $T_{sr}$ increases with decreasing T below LLE and decreases with increasing T above ULE , all in agreement with the numerical results of Figure 3a. Another study reports a similar trend in motor activity in rats under shortening LD cycles (Campuzano et al., 1998), as shown in Figure 3a (see black crosses). In this figure, we compared our results on the period of the second (dissociated) rhythm with the experimental data from Campuzano et al. (1998). Despite the clear separation between calculated and experimental curves, the trend is generally the same, and the relative difference between the numerical result and experiment is only 4% .

Second, as shown in Figure 3b, the entrainment range is characterized by a single frequency component of intensity $I_{F}^{\left(\upsilon \right)}=I_{F}^{\left(d\right)}$ in the core and shell. This component corresponds to an entrained rhythm with period T between LLE=23.26h (lower bound) and ULE=25.28h (upper bound), as shown in Figure 3a.

Third, analysis of the intensity of core and shell components reveals distinct contributions to the second rhythm above ULE and below LLE , as shown in Figure 3b. Below LLE , core and shell contributions to the second rhythm at period $T_{sr}$ increase, while contributions to the rhythm at period T decrease. However, when the LD period $T_{F}$ is lengthened above ULE , the core’s contribution is almost exclusively to the component at the LD period (near constant $I_{F}^{\left(\upsilon \right)}$ and vanishingly small $I_{sr}^{\left(\upsilon \right)}$ ), in qualitative agreement with experimental observations made by Schwartz et al. (2009). This difference is related to the coexistence of dynamically and topologically distinct states in the core and shell at T>ULE (these states were discussed in Yoon et al. (2021)). The core is on average locked to the LD cue, while the shell is drifting at the second rhythm. Below LLE , both the core and shell drift at the second rhythm. In this particular case (set of model parameters), the emergence of distinct states in the SCN is a consequence of distinct types of bifurcations in equations (4)-(7) at LLE and ULE . The bifurcation at LLE is characterized by the emergence of a second rhythm with period $T_{sr}∼∼T$ , as shown in Figure 3a. The bifurcation at ULE , however, is characterized by a large drop in $T_{sr}$ relative to T=ULE . These characteristic features of distinct bifurcations are also consistent with the experimental data discussed in the previous paragraph. For example, the data reported by Usui et al. (2000) show that $LLE-T_{sr}=0.5\;h$ , whereas $T_{sr}-ULE=3.5\;h$ for rats. For comparison, our model predicts $LLE-T_{sr}=0.2\;h$ and $T_{sr}-ULE=1.7\;h$ for the model parameters in Table 1. To identify the type of bifurcation, we also carried out the stability analysis of fixed points of equations (4)-(7). This analysis revealed that the dissociation at ULE=25.28h is caused by the super Hopf bifurcation, while the saddle-node bifurcation occurs at LLE=23.26h .

The results presented in this section are in good qualitative agreement with experimental observations of dissociation in the SCN. In the core-shell model of equations (4)-(7), the emergence of a dissociated rhythm takes place when entrainment is disrupted. The dynamical features and bifurcation mechanisms of disrupted states in the core and shell describe key aspects of dissociation, namely, the inverse dependence of the dissociated period on the LD period.

Notably, the upper and lower bounds of the entrainment range are very close to the mean free-running period of the core $\tau_{\upsilon }=25.1\;h$ and shell $\tau_{d}=23.3\;h$ for the model parameters in Table 1:

(14) $$\tau_{d}∼∼LLE<T<ULE∼∼\tau_{\upsilon }.$$

To determine the dependence of the entrainment range on the endogenous core and shell rhythms, we performed additional numerical calculations of equations (4)-(7) and analyzed the dependence of the entrainment range on the difference between the free-running periods, $\tau_{\upsilon }-\tau_{d}$ , at fixed $\tau_{\upsilon }$ and increasing $\tau_{d}$ . The results of these numerical calculations are presented in Figure 3c and show that decreasing the difference $\tau_{\upsilon }-\tau_{d}$ increases the entrainment range. Simultaneously, equation (13) shows that the difference between peaks in the core and shell, $t_{\upsilon }^{\left(p\right)}-t_{d}^{\left(p\right)}$ (anticipation), decreases with increasing $\tau_{d}$ (i.e., decreasing $\omega_{d}$ ). Therefore, one can have a broad entrainment range but weak anticipation, and vice versa.

### DD Conditions

This section studies the behavior of the core and shell under DD conditions and how core-shell intercouplings $K_{\upsilon d}$ and $K_{d\upsilon }$ are related to $\tau_{DD}$ across the whole SCN. Since $\tau_{DD}$ , $\tau_{\upsilon }$ , and $\tau_{d}$ can be measured experimentally under DD conditions, the aim is to identify any experimentally testable relationships that reveal information about the value and sign of intercouplings $K_{\upsilon d}$ and $K_{d\upsilon }$ .

The free-running frequency $\omega_{DD}=2\pi /\tau_{DD}$ of the synchronized SCN under DD conditions can be obtained self-consistently from the stationary state of equations (4)-(7), by setting F=0 and replacing $\omega_{F}\to \omega_{DD}$ , from which it follows that

(15) $$\omega_{DD}=\frac{a}{\left(a+b\right)}{\bar{\omega }}_{\upsilon }+\frac{b}{\left(a+b\right)}{\bar{\omega }}_{d},$$

where

(16) $$a=\rho_{\upsilon }^{2}K_{\upsilon d}(1+\rho_{d}^{2}),$$

(17) $$b=\rho_{d}^{2}K_{d\upsilon }(1+\rho_{\upsilon }^{2}).$$

The synchronized SCN is characterized by core and shell synchronization indices $\rho_{\upsilon }$ and $\rho_{d}$ . To find $\tau_{DD}$ , we solve numerically equations (4)-(7) at DD conditions with parameters in Table 1. Then we apply the Fourier analysis to the time dependence of real parts, Re[zυ(d)(t)]=ρυ(d)cos(ψυ(d)) , of the order parameters (equation (3)) of the core and shell. It gives the frequency of steady oscillations at DD conditions.

Motivated by the experimental observation in Taylor et al. (2017), we assume that $\tau_{\upsilon }>\tau_{d}$ (see Table 1). In the case for positive intercouplings $K_{\upsilon d}$ and $K_{d\upsilon }$ , the core-shell model of equations (4)-(7) predicts that ${\bar{\omega }}_{\upsilon }<\omega_{DD}<{\bar{\omega }}_{d}$ . Equivalently, the period $\tau_{DD}=2\pi /\omega_{DD}$ must be in the range

(18) $$\tau_{d}<\tau_{DD}<\tau_{\upsilon }.$$

Using the parameters in Table 1, equation (15) tells us that $\tau_{DD}=24.8\;h$ , in good agreement with experimental observations in rats by Campuzano et al. (1998), who reported $\tau_{DD}=24.5\;h$ .

Under DD conditions, the analytical result of equation (18) predicts that $\tau_{DD}$ is bounded between $\tau_{\upsilon }$ and $\tau_{d}$ . This is valid for positive inter- and intracouplings. However, in the case of negative couplings, $\tau_{DD}$ is no longer bound between $\tau_{\upsilon }$ and $\tau_{d}$ . In the particular case when $K_{d\upsilon }<0$ (and $K_{\upsilon d}\geq 0$ ), equation (15) becomes

(19) $$\omega_{DD}={\bar{\omega }}_{\upsilon }-\frac{\left|b|({\bar{\omega }}_{d}-{\bar{\omega }}_{\upsilon }\right)}{\left(a-|b|\right)}$$

from which it follows that $\tau_{DD}>\tau_{\upsilon }$ for ${\bar{\omega }}_{\upsilon }<{\bar{\omega }}_{d}$ ( $\tau_{\upsilon }>\tau_{d}$ ), under the additional assumption that $|K_{d\upsilon }|<K_{\upsilon d}$ as suggested by experimental observations (Varadarajan et al., 2018) for wild-type (WT) mice. Equation (19) also shows that strong asymmetry in intercoupling strength can cause the rhythm in the shell to become increasingly similar to the core, since $\tau_{DD}∼∼\tau_{\upsilon }$ when $|K_{d\upsilon }|\ll K_{\upsilon d}$ . Conversely, if $K_{d\upsilon }$ is instead positive and $K_{\upsilon d}$ negative, it can be shown that the rhythm in the core becomes increasingly similar to the shell for $|K_{d\upsilon }|\ll K_{\upsilon d}$ . This follows from re-writing equation (19) for $K_{\upsilon d}<0$ (and $K_{d\upsilon }\geq 0$ ) based on the same experimental observations ( $\tau_{\upsilon }>\tau_{d}$ and $K_{d\upsilon }<|K_{\upsilon d}|$ ), for which $\tau_{DD}<\tau_{d}$ . The results of equations (18) and (19) show that experimental measurements of $\tau_{DD}$ , $\tau_{\upsilon }$ , and $\tau_{d}$ can indicate whether $K_{d\upsilon }$ is positive or negative.

The relative strength of intercouplings $K_{\upsilon d}$ and $K_{d\upsilon }$ can also be estimated when the intracouplings $K_{\upsilon \upsilon }$ and $K_{dd}$ are large by comparison. Under these conditions, $\rho_{\upsilon }\approx \rho_{d}\approx 1$ , so that equation (15) takes the form

(20) $$\omega_{DD}=\frac{K_{\upsilon d}}{\left(K_{\upsilon d}+K_{d\upsilon }\right)}{\bar{\omega }}_{\upsilon }+\frac{K_{d\upsilon }}{\left(K_{\upsilon d}+K_{d\upsilon }\right)}{\bar{\omega }}_{d}.$$

This equation can be rearranged to yield the relative intercoupling strength

(21) $$\frac{K_{\upsilon d}}{K_{d\upsilon }}=\frac{\tau_{DD}/\tau_{d}-1}{1-\tau_{DD}/\tau_{\upsilon }},$$

which can therefore be determined from experimental values of $\tau_{DD}$ , $\tau_{\upsilon }$ , and $\tau_{d}$ .

A lower bound on the total intercoupling $\left|K_{\upsilon d}+K_{d\upsilon }\right|$ can be established with reference to the phase difference $\psi_{d}-\psi_{\upsilon }$ under DD conditions. From equation (9) under LD conditions, the replacement $\omega_{F}\to \omega_{DD}$ yields

(22) $$\sin(\psi_{d}-\psi_{\upsilon })=\frac{2\rho_{d}({\bar{\omega }}_{d}-\omega_{DD})}{\rho_{\upsilon }K_{\upsilon d}(1+\rho_{d}^{2})}=\frac{2\rho_{\upsilon }\rho_{d}({\bar{\omega }}_{d}-{\bar{\omega }}_{\upsilon })}{\left(a+b\right)}.$$

First, this shows that $\psi_{d}$ leads $\psi_{\upsilon }$ under DD conditions, as experimental observations indicate that $\tau_{\upsilon }>\tau_{d}$ ( ${\bar{\omega }}_{\upsilon }<{\bar{\omega }}_{d}$ ) and $K_{d\upsilon }<K_{\upsilon d}$ (Taylor et al., 2017; Varadarajan et al., 2018), assuming positive intercoupling. In this case, peak activity in the shell precedes peak activity in the core, similar to LD conditions (see equation (12)). However, if either $K_{\upsilon d}$ or $K_{d\upsilon }$ becomes negative, their relative strength can cause the core to lead the shell under DD conditions, that is, if $K_{\upsilon d}\gg K_{d\upsilon }$ or $K_{d\upsilon }\gg K_{\upsilon d}$ . For the same to take place under LD conditions, $K_{\upsilon d}$ must simply be negative. Second, when the intracouplings are sufficiently larger than the intercouplings, $\rho_{\upsilon }\approx \rho_{d}\approx 1$ , so that $a∼∼2K_{\upsilon d}$ and $b∼∼2K_{d\upsilon }$ ; equation (22) then becomes

(23) $$\sin(\psi_{d}-\psi_{\upsilon })\approx \frac{{\bar{\omega }}_{d}-{\bar{\omega }}_{\upsilon }}{K_{\upsilon d}+K_{d\upsilon }}.$$

Thus, the total intercoupling must be larger than or equal to the difference between the mean natural frequencies of the shell and core, in absolute value:

(24) $$|K_{\upsilon d}+K_{d\upsilon }|\geq |{\bar{\omega }}_{d}-{\bar{\omega }}_{\upsilon }|,$$

since $|\sin(\psi_{d}-\psi_{\upsilon })|\leq 1$ .

The results of this section show that the sign, relative strength, and the minimum total intercoupling can be determined from the free-running periods of the core ( $\tau_{\upsilon }$ ), shell ( $\tau_{d}$ ), and the SCN as a whole ( $\tau_{DD}$ ), which can be measured experimentally under DD conditions. Moreover, the results show that, if all other parameters remain the same, changes in the relative strength and sign of the intercouplings can cause the core to lead the shell even when $\tau_{\upsilon }>\tau_{d}$ , and cause the synchronized rhythm of the core to approximate the endogenous (free-running) rhythm of the shell or vice versa. Below the minimum total intercoupling $\left|K_{\upsilon d}+K_{d\upsilon }|=|{\bar{\omega }}_{d}-{\bar{\omega }}_{\upsilon }\right|$ , the SCN is no longer able to synchronize.

### LL Conditions

This section studies the SCN under constant light or LL conditions for diurnal and nocturnal animals. Within our approach, LL conditions can be seen as the limit when LD cycles become so slow that they are effectively constant from the point of view of individual oscillators in the core. In turn, this motivates the replacement of the periodic forcing term in equation (2) with a constant B :

(25) $$F\sin(\omega_{F}t+\phi -\theta_{i}^{\left(\upsilon \right)})\to B.$$

In our reduced Kuramoto model, parameter B is phenomenological. It can be positive or negative. We assume that the magnitude |B| characterizes light intensity and chooses the sign of B to satisfy Aschoff’s first rule. From this, it follows that B is negative for nocturnal animals and positive for diurnal animals, as will be proven below. This choice of parameter B allows us to describe the basic properties of the SCN under LL conditions, in nocturnal and diurnal animals.

As one can see in equation (2), introducing parameter B renormalizes the endogenous free-running frequencies of core oscillators

(26) $$\omega_{i}^{\left(\upsilon \right)}\Rightarrow \omega_{i}^{\left(\upsilon \right)}+B,$$

from which it follows that the renormalized mean frequency of the core is

(27) $${\bar{\omega }}_{\upsilon }^{*}={\bar{\omega }}_{\upsilon }+B,$$

and, equivalently, the renormalized mean period of oscillations is

(28) $$\tau_{\upsilon }^{*}=\frac{2\pi }{{\bar{\omega }}_{\upsilon }^{*}}=\frac{\tau_{\upsilon }}{1+\frac{B\tau_{\upsilon }}{2\pi }},$$

where $\tau_{\upsilon }$ is the endogenous mean free-running period of isolated core oscillators. This shows that under LL conditions, the positive parameter B>0 (diurnal animals) forces core oscillators to run faster than under DD conditions ( ${\bar{\omega }}_{\upsilon }^{*}>{\bar{\omega }}_{\upsilon }$ ). Conversely, if parameter B is negative, $-2{\bar{\omega }}_{\upsilon }<B<0$ , (nocturnal animals), then the constant light cue forces core oscillators to run slower than under DD conditions ( ${\bar{\omega }}_{\upsilon }^{*}<{\bar{\omega }}_{\upsilon }$ ). The free-running period of isolated shell oscillators, on the other hand, remains unaffected ( $\tau_{d}^{*}=\tau_{d}$ ), since it is not directly exposed to the light cue. It would be interesting to check experimentally this effect of LL conditions on isolated clock cells in the core.

To determine $\tau_{LL}$ in our core-shell model, we must account for the intercoupling between the core and shell. The dynamics of the core-shell system under LL conditions are described by the same equations (4)-(7) as DD conditions ( F=0 ), by renormalizing the mean period as in equation (28). This means that we can use the equations derived for DD conditions by making the simple replacements $\tau_{\upsilon }\to \tau_{\upsilon }^{*}$ and $\tau_{DD}\to \tau_{LL}$ . From equation (20) in particular, it then follows that the frequency $\omega_{LL}$ of the steady circadian rhythm is

(29) $$\begin{array}{l} \omega_{LL}=\frac{a}{\left(a+b\right)}{\bar{\omega }}_{\upsilon }^{*}+\frac{b}{\left(a+b\right)}{\bar{\omega }}_{d}\\ \;=\frac{a}{\left(a+b\right)}({\bar{\omega }}_{\upsilon }+B)+\frac{b}{\left(a+b\right)}{\bar{\omega }}_{d}.\; \end{array}$$

Based on experimental observations of different animal species, Aschoff’s first rule predicts that $\tau_{LL}<\tau_{DD}$ in diurnal animals and $\tau_{LL}>\tau_{DD}$ in nocturnal animals, where $\tau_{DD}$ and $\tau_{LL}$ are the free-running circadian periods under DD and LL conditions, respectively (Aschoff, 1965, 1979; Pittendrigh and Daan, 1976a; Carpenter and Grossberg, 1984). Within our model, by comparison with equation (20), it follows that $\omega_{LL}-\omega_{DD}=aB{\bar{\omega }}_{\upsilon }/(a+b)$ , which satisfies Aschoff’s first rule when a/(a+b)>0 (refer to the discussion on the sign and strength of the intercouplings in the section on DD conditions). Assuming that B is positive for diurnal animals yields $\tau_{LL}<\tau_{DD}$ ( $\omega_{LL}>\omega_{DD}$ ). Conversely, negative B yields $\tau_{LL}>\tau_{DD}$ ( $\omega_{LL}<\omega_{DD}$ ) in nocturnal animals. Moreover, Aschoff’s first rule also states that the effects of LL conditions are intensity-dependent (Aschoff, 1965; Pittendrigh and Daan, 1976a; Carpenter and Grossberg, 1984). The free-running period of circadian activity rhythms usually decreases with increasing light intensity: the brighter the constant light, the faster the animal’s clock runs. In nocturnal animals, the converse is usually observed: the free-running period of the rhythm increases with increasing light intensity. Within the model of equation (25) and equations (4)-(7), the parameter |B| characterizes the intensity of the optic cue. For diurnal animals ( B>0 ), increasing |B| decreases $\tau_{LL}$ (increases $\omega_{LL}$ ), whereas for nocturnal animals ( B<0 ), increasing |B| increases $\tau_{LL}$ (decreases $\omega_{LL}$ ), in agreement with Aschoff’s first rule.

Next, this section explores the possibility of dissociation under increasing light intensity. To this end, the spectral analysis of the section on dissociation under LD conditions was repeated after numerically solving the model of equation (25) and equations (4)-(7), using the parameters in Table 1. These parameters were determined in reference to experimental observations of mice, which are nocturnal ( B<0 ). The case B>0 in Figure 4 describes a “fictitious diurnal mouse”. We use the notion “fictitious diurnal mouse” to outline that parameter B is positive for diurnal animals, although the remaining model parameters (Table 1) used throughout this article are for nocturnal mice. The result presented in Figure 4 shows that LL conditions slow down the circadian clock (i.e., $\tau_{LL}$ is lengthened), shortens the daily active phase, and reduces total daily activity in nocturnal rodents. In diurnal animals, including humans, LL conditions generally have the opposite effect (Mistlberger and Rusak, 2005), although there are some exceptions across species (diurnal primates in particular).

Spectral analysis revealed the emergence of a second rhythm with period $T_{LL}^{\left(sr\right)}$ at a critical brightness $\left|B_{c}\right|$ , with an order of magnitude difference between nocturnal and diurnal cases. In the nocturnal case, $|B_{c}|≃0.24$ , for which $\tau_{LL}=25.2\;h$ , whereas in the diurnal case, $|B_{c}|≃3.23$ , for which $\tau_{LL}=21.7\;h$ . These results are in qualitative agreement with the experimental observation of rhythm dissociation at constant dim illumination of approximately 4.5 lux in rats for which $\tau_{LL}=25.15\;h$ following 3 months of exposure (Albers et al., 1981). Unfortunately, due to a lack of detailed experimental data, we are unable to present a detailed numerical comparison as a function of illumination. However, we expect that the dependence of dissociated rhythm period, $T_{LL}^{\left(sr\right)}$ , on light intensity |B| for diurnal and nocturnal animals can be checked experimentally. In addition, Figure 4 shows that with increasing the light intensity, |B| , the period of the dissociated rhythm decreases a little in the nocturnal case but slightly increases in the diurnal case. Unlike the free-running period $\tau_{LL}$ , the dissociated rhythm $T_{LL}^{\left(sr\right)}$ does not obey Aschoff’s first rule. Finally, the model of equation (25) and equations (4)-(7) predicts that the light intensity, |B| , can also alter the core-shell phase difference under LL conditions compared with DD conditions. Under LL conditions, the phase difference $\psi_{d}-\psi_{\upsilon }$ can be determined from

(30) $$\sin(\psi_{d}-\psi_{\upsilon })=\frac{2\rho_{d}({\bar{\omega }}_{d}-\omega_{LL})}{\rho_{\upsilon }K_{\upsilon d}(1+\rho_{d}^{2})}=\frac{2\rho_{\upsilon }\rho_{\upsilon }({\bar{\omega }}_{d}-{\bar{\omega }}_{\upsilon }-B)}{\left(a+b\right)},$$

which follows from the replacement $\omega_{\upsilon }\to \omega_{\upsilon }^{*}$ in equation ( 22 ). For diurnal animals ( B>0 ), increasing the light intensity decreases the core-shell phase difference in animals where $\tau_{d}<\tau_{\upsilon }$ and both intercouplings are positive. In nocturnal animals, increasing the light intensity increases the core-shell phase difference. In both cases, sufficiently large light intensity, |B| , will invert the core-shell phase relationship, causing the core to lead the shell. Under these conditions, the shell cannot provide a reference phase for anticipation in peripheral clocks.

## Discussion

In this work, we studied how the core-shell organization controls the behavior of the SCN, the entrainment of the core and shell to the environmental cue, the mechanism of disruption of the synchronized state, and the impact of different light conditions on dynamics of the SCN. For this purpose, we used a core-shell model of the SCN based on reduced dynamical equations for the forced Kuramoto model. Our approach is based on the observation that clock cells in the SCN are self-sustained, nearly sinusoidal oscillators with a stable limit cycle. The first benefit of our model is that it only has 9 biologically meaningful parameters, instead of thousands of parameters for an equally large number of neurons. Second, the proposed dynamical equations for core and shell rhythms describe known SCN activity under 3 lighting conditions (LD, DD, and LL), including the anticipation and the dissociation of the circadian rhythms in the dependence on the period of external optic cue and light intensity. Third, the model can be calibrated for diurnal and nocturnal mammals. In this article, we calibrated the model by using parameters for mice. Because these nocturnal animals are good model animals, there are numerous experimental studies on the behavior of their circadian rhythms under different light conditions, and reliable quantitative and qualitative results were obtained. Fourth, since our model is based on explicit equations, it allows us to perform both an analytical analysis and detailed numerical comparison between the experiment and theory concerning the temporal behavior of the SCN as a whole and its principal modules, the core and shell, at different light conditions, in the entrained state and the states with dissociated rhythms. As far as we know, this detailed comparison was not performed within the existing models.

An important difference between the core and shell is that neuron populations within each subdivision have distinct mean free-running periods $\tau_{\upsilon }$ and $\tau_{d}$ , respectively. The numerical results present in Figure 3 suggest that $\tau_{\upsilon }$ and $\tau_{d}$ are important parameters that determine the entrainment range and the core-shell phase difference under LD conditions. From an evolutionary point of view, this suggests that the differentiation between endogenous rhythms in the core and shell is an adaptation to environmental variations in LD cycle length.

Under DD conditions, the analytical results of equation (18) predict that the SCN’s free-running period $\tau_{DD}$ is bounded between $\tau_{\upsilon }$ and $\tau_{d}$ . Based on experimental evidence that $\tau_{d}<24\;h$ and $\tau_{\upsilon }>24\;h$ (Taylor et al., 2017), equation (18) predicts that the core-shell SCN can maintain near-24-h periods of activity in the absence of an external light cue. Our numerical result, $\tau_{DD}=24.8\;h$ , obtained for the model parameters calibrated for mice, agrees with Campuzano et al. (1998), who reported $\tau_{DD}=24.5\;h$ for rats. From a biological point of view, the ability to retain circadian activity in constant darkness ensures minimum disruption of circadian activity. However, this ability is also dependent on the relative strength of core-shell communication, parametrized by intercouplings $K_{\upsilon d}$ and $K_{d\upsilon }$ . As discussed in the section on DD conditions, a large asymmetry in the strength of the intercouplings, which parametrize core-shell communication, is expected to shift the free-running period under constant darkness closer to the endogenous rhythm of the core ( $K_{\upsilon d}\gg K_{d\upsilon }$ ) or the shell ( $K_{d\upsilon }\gg K_{\upsilon d}$ ).

The period of an external cue (LD cycles) as the control parameter can be easily adjusted in laboratory conditions as in many experimental investigations. Here, we demonstrated appearance of dissociation when the period of the LD cycle is larger (or smaller) than a critical value. There are also other important parameters, such as couplings between oscillators and mean free-running periods in the core and shell. Changes of these parameters also may result in the dissociation of circadian rhythms. The impact of abnormal changes of these parameters on the SCN dynamics is an important problem in experimental research. External factors, such as temperature or continuous intake of chemical substances, for example, melatonin or antidepressants, may change these parameters. In particular, the couplings among neurons are mediated by neurotransmitters, such as VIP and GABA, which can be influenced by drugs. The appearance of dissociated rhythms is a common phenomenon in the SCN of mammals. We believe that our results may also be translated to humans being diurnal animals. However, these interesting problems need a detailed literature search and further investigations that go out of the scope of the present article.

Our model shows that the SCN’s functional separation into a core and shell enables anticipation, the ability of an organism to trigger physiological changes and behavior in anticipation of regular events. As argued in the section on anticipation in the entrained state, anticipation is supported by the core-shell phase difference $\psi_{d}-\psi_{\upsilon }$ , which is proportional to the difference in peak activity time between the shell and the core. Experimental evidence shows that the shell coordinates the phase of tissue clocks throughout the brain and body (Evans et al., 2015; Coomans et al., 2015; Silver, 2018; Gizowski et al., 2016), and peak activity in the shell precedes peak activity in the core (Taylor et al., 2017) under LD conditions. This corresponds to a situation where the shell phase $\psi_{d}$ leads the core phase, $\psi_{\upsilon }$ , which is locked to the cue phase and acts as a reference cue for anticipation in peripheral clocks. Under LD cycles with angular frequency $\omega_{F}=2\pi /T$ , the core-shell phase difference is determined by the relative magnitude and sign of intercoupling $K_{\upsilon d}$ and the shell detuning ${\bar{\omega }}_{d}-\omega_{F}$ . In this case, the shell leads the core provided that communication from the core has a synchronizing effect ( $K_{\upsilon d}>0$ ). For a given LD cycle with period T , the extent of anticipation (phase difference) varies inversely with the strength of $K_{\upsilon d}$ . Under DD conditions, anticipation becomes dependent on the relative magnitude and sign of the difference $\tau_{\upsilon }-\tau_{d}$ and the total intercoupling $K_{\upsilon d}+K_{d\upsilon }$ , as shown in the section on DD conditions. In this case, the shell phase can also precede the phase in the core, even when one of the intercouplings has a weak desynchronizing effect, and the extent of anticipation varies inversely with the total intercoupling.

We also considered the behavior of the SCN under LL condition by introducing a phenomenological parameter B which can be positive or negative. Its magnitude |B| characterizes the light intensity. By choosing the sign of B to satisfy Aschoff’s first rule, negative B for nocturnal animals and positive B for diurnal animals, we described the basic properties of the SCN under LL conditions for nocturnal and diurnal animals in qualitative and quantitative agreements with observations. Compared to DD conditions, the analytical results for LL conditions predict that a constant light cue with intensity |B| decreases the core-shell phase difference in diurnal animals ( B>0 ) but has the opposite effect in nocturnal animals ( B<0 ). However, sufficiently large light intensity causes the shell to lag the core and is therefore expected to make anticipation impossible in both nocturnal and diurnal animals.

At the present time, there is surprisingly little research aimed specifically at determining the physiological, anatomical, or molecular mechanisms underlying Aschoff’s rules. Genetic components of Aschoff’s first rule were discussed by Muñoz et al. (2005). These authors found that LL lengthens the circadian period by inhibiting the normal dark-induced degradation of mPER2, and constitutively elevated levels of mPER2 act to enhance the phase-delaying limb of the molecular oscillator. Unfortunately, it is unclear how this inhibition-enhancement mechanism may relate to the sign of the phenomenological parameter B . It is an open problem for further experimental and theoretical investigations.

In this work, we also analyzed the difference in the collective behavior of the SCN oscillators under long and short photoperiod conditions. Operating limits on free-running and entrained SCN activity are partly determined by the same parameters as the core-shell phase difference $\psi_{d}-\psi_{\upsilon }$ . During steady-state activity, $0<|\psi_{d}-\psi_{\upsilon }|\leq \pi$ , as can be inferred from the parameters in Table 1 and equations (9), (22), and (30). The numerical results of Figures 3 (LD conditions) and 4 (LL conditions) predict rhythm dissociation under varying LD period or the light intensity under LL. Under LD conditions, entrainment is ensured for any LD period T within the entrainment range LLE<T<ULE . Outside this range, a second rhythm appears, and its period $T_{sr}$ varies inversely with the LD period. At ULE , $T_{sr}$ is significantly smaller than T and decreases with increasing T . At LLE , $T_{sr}$ is infinitesimally larger than T and increases with decreasing T . These results are in good qualitative agreement with experimental observations of dissociation in rats (Campuzano et al., 1998; Usui et al., 2000). Interestingly, the spectral analysis presented in Figure 3b predicts that the dissociated rhythm at ULE is largely produced by the shell, unlike at LLE , where the core and shell contribute almost equally to the dissociated rhythm. Under LL conditions, dissociation appears under increasing light intensity |B| in both diurnal ( B>0 ) and nocturnal animals ( B<0 ), as shown in Figure 4. The period $T_{LL}^{\left(sr\right)}$ of the dissociated rhythm is predicted to increase with light intensity in diurnal animals, but to decrease in nocturnal animals. In addition, the critical light intensity for dissociation was found to be an order of magnitude smaller in the nocturnal case, in agreement with the experimental observation of rhythm dissociation in rats under LL conditions (Albers et al., 1981).

In conclusion, the simplified model presented in this work captures important functional aspects of the SCN and its core-shell organization. Using this model, we extend existing work on dissociation by studying the period and strength of the dissociated rhythm. Numerical results were found to be in good qualitative agreement with experimental observations, in particular regarding the inverse relationship between the period of the LD cycle and the period of the dissociated rhythm. Within the model, the state of the core and shell is characterized by a synchronization index and phase, and determined by parameters which can either be measured experimentally or be inferred from experiments, as discussed in the section on the choice of parameter values. Among these parameters, the core-shell intercouplings and mean free-running periods constrain the core-shell phase difference, which determines the shell’s ability to anticipate the core under different symmetric lighting conditions and intensities. In the particular case of constant lighting conditions, the dependence on light intensity can be modeled explicitly through the introduction of a phenomenological constant, as shown in the corresponding section. This simple modification to the model reproduces Aschoff’s first rule for diurnal and nocturnal animals and reveals rhythm dissociation under increasing light intensity. Moreover, the introduction of the constant opens up the possibility to explore other open problems and experimental conditions in the study of circadian rhythms. Potential applications include asymmetric LD cycles, which are used to mimic seasonal variations in day length (Meijer et al., 2010; Myung et al., 2015, and the problem of antiphase rhythm splitting under constant light (Pittendrigh, 1960; Pittendrigh and Daan, 1976b; Ohta et al., 2005). From a theoretical point of view, another interesting problem is to extend the proposed model with other features of the SCN’s network organization, namely, degree distribution (Gu et al., 2021), the existence of local groups of neurons that form intermediate structures in the SCN (Yoshikawa et al., 2021).

The results presented in this work strongly suggest that the Kuramoto model captures essential features of synchronization and entrainment in the SCN. Moreover, the reduced model is easily extendible to an arbitrary number of groups, with dynamics described by explicit equations for the group phase and synchronization index. Viewed together, the reduced Kuramoto model presents itself as a useful tool for exploring open problems in the study of circadian rhythms, one that can account for evolving views of the circadian system’s organization, including inter-hemispheric interaction and peripheral clocks (e.g., in liver, muscle, pancreas, heart, adipose tissue). We believe that our model may be translated to the other animals, both nocturnal and diurnal animals, including humans.

## References

[bibr1-07487304221107834] Abrahamson EE Moore RY (2001) Suprachiasmatic nucleus in the mouse: retinal innervation, intrinsic organization and efferent projections. Brain Res 916:172-191. 11597605 10.1016/s0006-8993(01)02890-6

[bibr2-07487304221107834] Albers HE Gerall AA Axelson JF (1981) Circadian rhythm dissociation in the rat: effects of long-term constant illumination. Neurosci Lett 25:89-94. 7279304 10.1016/0304-3940(81)90106-3

[bibr3-07487304221107834] Aschoff J (1965) Circadian rhythms in man. Science 148:1427-1432. 14294139 10.1126/science.148.3676.1427

[bibr4-07487304221107834] Aschoff J (1979) Circadian rhythms: influences of internal and external factors on the period measured in constant conditions. Z Tierpsychol 49:225-249. 386643 10.1111/j.1439-0310.1979.tb00290.x

[bibr5-07487304221107834] Campuzano A Vilaplana J Cambras T Díez-Noguera A (1998) Dissociation of the rat motor activity rhythm under t cycles shorter than 24 hours. Physiol Behav 63:171-176. 9423955 10.1016/s0031-9384(97)00416-2

[bibr6-07487304221107834] Carpenter GA Grossberg S (1984) A neural theory of circadian rhythms: Aschoff’s rule in diurnal and nocturnal mammals. Am J Physiol 247:R1067-R1082. 6542316 10.1152/ajpregu.1984.247.6.R1067

[bibr7-07487304221107834] Childs LM Strogatz SH (2008) Stability diagram for the forced Kuramoto model. Chaos 18:043128. 19123638 10.1063/1.3049136

[bibr8-07487304221107834] Coomans CP Ramkisoensing A Meijer JH (2015) The suprachiasmatic nuclei as a seasonal clock. Front Neuroendocrinol 37:29-42. 25451984 10.1016/j.yfrne.2014.11.002

[bibr9-07487304221107834] Daan S Berde C (1978) Two coupled oscillators: simulations of the circadian pacemaker in mammalian activity rhythms. J Theor Biol 70:297-313. 633922 10.1016/0022-5193(78)90378-8

[bibr10-07487304221107834] de la Iglesia HO Cambras T Schwartz WJ Díez-Noguera A (2004) Forced desynchronization of dual circadian oscillators within the rat suprachiasmatic nucleus. Curr Biol 14:796-800. 15120072 10.1016/j.cub.2004.04.034

[bibr11-07487304221107834] de la Iglesia HO Meyer J Carpino A Schwartz WJ (2000) Antiphase oscillation of the left and right suprachiasmatic nuclei. Science 290:799-801. 11052942 10.1126/science.290.5492.799

[bibr12-07487304221107834] Dorogovtsev SN Goltsev AV Mendes JF (2008) Critical phenomena in complex networks. Rev Mod Phys 80:1275.

[bibr13-07487304221107834] Evans JA Gorman MR (2016) In synch but not in step: circadian clock circuits regulating plasticity in daily rhythms. Neuroscience 320:259-280. 26861419 10.1016/j.neuroscience.2016.01.072 PMC4793422

[bibr14-07487304221107834] Evans JA Elliott JA Gorman MR (2005) Circadian entrainment and phase resetting differ markedly under dimly illuminated versus completely dark nights. Behav Brain Res 162:116-126. 15922072 10.1016/j.bbr.2005.03.014

[bibr15-07487304221107834] Evans JA Leise TL Castanon-Cervantes O Davidson AJ (2013) Dynamic interactions mediated by nonredundant signaling mechanisms couple circadian clock neurons. Neuron 80:973-983. 24267653 10.1016/j.neuron.2013.08.022 PMC3841113

[bibr16-07487304221107834] Evans JA Suen TC Callif BL Mitchell AS Castanon-Cervantes O Baker KM Kloehn I Baba K Teubner BJ Ehlen JC , et al. (2015) Shell neurons of the master circadian clock coordinate the phase of tissue clocks throughout the brain and body. BMC Biology 13:43. 26099272 10.1186/s12915-015-0157-x PMC4489020

[bibr17-07487304221107834] Gizowski C Zaelzer C Bourque C (2016) Clock-driven vasopressin neurotransmission mediates anticipatory thirst prior to sleep. Nature 537:685-688. 27680940 10.1038/nature19756

[bibr18-07487304221107834] Goldbeter A Leloup JC (2021) From circadian clock mechanism to sleep disorders and jet lag: insights from a computational approach. Biochem Pharmacol 191:114482. 33617843 10.1016/j.bcp.2021.114482

[bibr19-07487304221107834] Gu C Li J Zhou J Yang H Rohling J (2021) Network structure of the master clock is important for its primary function. Front Physiol 12:678391. 34483953 10.3389/fphys.2021.678391 PMC8415478

[bibr20-07487304221107834] Gu C Tang M Yang H (2016) The synchronization of neuronal oscillators determined by the directed network structure of the suprachiasmatic nucleus under different photoperiods. Sci Rep 6:28878. 27358024 10.1038/srep28878 PMC4928114

[bibr21-07487304221107834] Gu C Yang H Meijer JH Rohling JH (2018) Dependence of the entrainment on the ratio of amplitudes between two subgroups in the suprachiasmatic nucleus. Physical Review E 97:062215. 30011551 10.1103/PhysRevE.97.062215

[bibr22-07487304221107834] Güldner FH (1976) Synaptology of the rat suprachiasmatic nucleus. Cell Tissue Res 165:509-544. 1260842 10.1007/BF00224478

[bibr23-07487304221107834] Hafner M Koeppl H Gonze D (2012) Effect of network architecture on synchronization and entrainment properties of the circadian oscillations in the suprachiasmatic nucleus. PLoS Comput Biol 8:e1002419. 22423219 10.1371/journal.pcbi.1002419 PMC3297560

[bibr24-07487304221107834] Hannay KM Forger DB Booth V (2020) Seasonality and light phase-resetting in the mammalian circadian rhythm. Sci Rep 10:19506. 33177530 10.1038/s41598-020-74002-2 PMC7658258

[bibr25-07487304221107834] Hastings MH Maywood ES Brancaccio M (2018) Generation of circadian rhythms in the suprachiasmatic nucleus. Nat Rev Neurosci 19:453-469. 29934559 10.1038/s41583-018-0026-z

[bibr26-07487304221107834] Ko CH Takahashi JS (2006) Molecular components of the mammalian circadian clock. Hum Mol Genet 15:R271-R277. 16987893 10.1093/hmg/ddl207

[bibr27-07487304221107834] Leak RK Card JP Moore RY (1999) Suprachiasmatic pacemaker organization analyzed by viral transynaptic transport. Brain Res 819:23-32. 10082857 10.1016/s0006-8993(98)01317-1

[bibr28-07487304221107834] Leloup JC Goldbeter A (2003) Toward a detailed computational model for the mammalian circadian clock. Proc Natl Acad Sci U S A 100:7051-7056. 12775757 10.1073/pnas.1132112100 PMC165828

[bibr29-07487304221107834] Liu C Weaver DR Strogatz SH Reppert SM (1997) Cellular construction of a circadian clock: period determination in the suprachiasmatic nuclei. Cell 91:855-860. 9413994 10.1016/s0092-8674(00)80473-0

[bibr30-07487304221107834] Lu Z Klein-Cardeña K Lee S Antonsen TM Girvan M Ott E (2016) Resynchronization of circadian oscillators and the east-west asymmetry of jet-lag. Chaos 26:094811. 27781473 10.1063/1.4954275

[bibr31-07487304221107834] Meijer JH Groos GA Rusak B (1986) Luminance coding in a circadian pacemaker: the suprachiasmatic nucleus of the rat and the hamster. Brain Res 382:109-118. 3768668 10.1016/0006-8993(86)90117-4

[bibr32-07487304221107834] Meijer JH Michel S VanderLeest HT Rohling JH (2010) Daily and seasonal adaptation of the circadian clock requires plasticity of the SCN neuronal network. Eur J Neurosci 32:2143-2151. 21143668 10.1111/j.1460-9568.2010.07522.x

[bibr33-07487304221107834] Michel S Meijer JH (2020) From clock to functional pacemaker. Eur J Neurosci 51:482-493. 30793396 10.1111/ejn.14388 PMC7027845

[bibr34-07487304221107834] Michel S Marek R Vanderleest HT Vansteensel MJ Schwartz WJ Colwell CS Meijer JH (2013) Mechanism of bilateral communication in the suprachiasmatic nucleus. Eur J Neurosci 37:964-971. 23311402 10.1111/ejn.12109

[bibr35-07487304221107834] Mistlberger RE Rusak B (2011) Circadian rhythms in mammals: formal properties and environmental influences. In: Kryger MH Roth T Dement WC editors. Principles and practice of sleep medicine. 5th ed. Elsevier, p. 363-375.

[bibr36-07487304221107834] Moore RY (1996) Entrainment pathways and the functional organization of the circadian system. Prog Brain Res 111:103-119. 8990910 10.1016/s0079-6123(08)60403-3

[bibr37-07487304221107834] Moore RY Bernstein ME (1989) Synaptogenesis in the rat suprachiasmatic nucleus demonstrated by electron microscopy and synapsin I immunoreactivity. J Neurosci 9:2151-2162. 2498469 10.1523/JNEUROSCI.09-06-02151.1989 PMC6569714

[bibr38-07487304221107834] Muñoz M Peirson SN Hankins MW Foster RG (2005) Long-term constant light induces constitutive elevated expression of mPER2 protein in the murine SCN: a molecular basis for Aschoff’s rule? J Biol Rhythms 20:3-14. 15654066 10.1177/0748730404272858

[bibr39-07487304221107834] Myung J Hong S DeWoskin D DeSchutter E Forger D Takumi T (2015) GABA-mediated repulsive coupling between circadian clock neurons in the SCN encodes seasonal time. Proc Natl Acad Sci U S A 112:E3920-E3929. 26130804 10.1073/pnas.1421200112 PMC4517217

[bibr40-07487304221107834] Noguchi T Watanabe K Ogura A Yamaoka S (2004) The clock in the dorsal suprachiasmatic nucleus runs faster than that in the ventral. Eur J Neurosci 20:3199-3202. 15579176 10.1111/j.1460-9568.2004.03784.x

[bibr41-07487304221107834] Oda GA Friesen WO (2002) A model for “splitting” of running-wheel activity in hamsters. J Biol Rhythms 17:76-88. 11837951 10.1177/074873002129002357

[bibr42-07487304221107834] Ohta H Yamazaki S McMahon DG (2005) Constant light desynchronizes mammalian clock neurons. Nat Neurosci 8:267-269. 15746913 10.1038/nn1395

[bibr43-07487304221107834] Ott E Antonsen TM (2008) Low dimensional behavior of large systems of globally coupled oscillators. Chaos 18:037113. 19045487 10.1063/1.2930766

[bibr44-07487304221107834] Pauls S Foley N Foley D LeSauter J Hastings M Maywood E Silver R (2014) Differential contributions of intra-cellular and inter-cellular mechanisms to the spatial and temporal architecture of the suprachiasmatic nucleus circadian circuitry in wild-type, cryptochrome-null and vasoactive intestinal peptide receptor 2-null mutant mice. Eur J Neurosci 40:2528-2540. 24891292 10.1111/ejn.12631 PMC4159586

[bibr45-07487304221107834] Pittendrigh CS (1960) Circadian rhythms and the circadian organization of living systems. Cold Spring Harb Symp Quant Biol 25:159-184. 10.1101/sqb.1960.025.01.015 13736116

[bibr46-07487304221107834] Pittendrigh CS Daan S (1976a) A functional analysis of circadian pacemakers in nocturnal rodents. J Comp Physiol 106:291-331.

[bibr47-07487304221107834] Pittendrigh CS Daan S (1976b) A functional analysis of circadian pacemakers in nocturnal rodents. J Comp Physiol 106:333-355.

[bibr48-07487304221107834] Pittendrigh CS Daan S (1976c) A functional analysis of circadian pacemakers in nocturnal rodents. IV. Entrainment: pacemaker as clock. J Comp Physiol 106:291-331.

[bibr49-07487304221107834] Rohling JH Meylahn JM (2020) Two-community noisy Kuramoto model suggests mechanism for splitting in the suprachiasmatic nucleus. J Biol Rhythms 35:158-166. 31969025 10.1177/0748730419898314 PMC7031819

[bibr50-07487304221107834] Rohr KE Pancholi H Haider S Karow C Modert D Raddatz NJ Evans J (2019) Seasonal plasticity in GABAA signaling is necessary for restoring phase synchrony in the master circadian clock network. eLife 8:e49578. 31746738 10.7554/eLife.49578 PMC6867713

[bibr51-07487304221107834] Schibler U Gotic I Saini C Gos P Curie T Emmenegger Y Sinturel F Gosselin P Gerber A Fleury-Olela F , et al. (2015) Clock-talk: interactions between central and peripheral circadian oscillators in mammals. Cold Spring Harb Symp Quant Biol 80:223-232. 10.1101/sqb.2015.80.027490 26683231

[bibr52-07487304221107834] Schwartz MD Wotus C Liu T Friesen WO Borjigin J Oda GA Horacio O (2009) Dissociation of circadian and light inhibition of melatonin release through forced desynchronization in the rat. Proc Natl Acad Sci U S A 106:17540-17545. 19805128 10.1073/pnas.0906382106 PMC2762670

[bibr53-07487304221107834] Silver R Taub A Li A (2018) Suprachiasmatic nucleus anatomy, physiology, and neurochemistry. oxford research encyclopedia of neuroscience: neuroendocrine and autonomic systems. In: Nelson R editor. Print encyclopedia as part of larger digital project, headed by Editor in Chief Murray Sherman. Oxford University Press.

[bibr54-07487304221107834] Taylor SR Wang TJ Granados-Fuentes D Herzog ED (2017) Resynchronization dynamics reveal that the ventral entrains the dorsal suprachiasmatic nucleus. J Biol Rhythms 32:35-47. 28326909 10.1177/0748730416680904 PMC5483321

[bibr55-07487304221107834] Tokuda IT Ono D Honma S Honma KI Herzel H (2018) Coherency of circadian rhythms in the SCN is governed by the interplay of two coupling factors. PLoS Comput Biol 14:e1006607. 30532130 10.1371/journal.pcbi.1006607 PMC6301697

[bibr56-07487304221107834] Usui S Takahashi Y Okazaki T (2000) Range of entrainment of rat circadian rhythms to sinusoidal light-intensity cycles. Am J Physiol 278:R1148-R1156. 10.1152/ajpregu.2000.278.5.R1148 10801281

[bibr57-07487304221107834] Varadarajan S Tajiri M Jain R Holt R Ahmed Q LeSauter J Silver R (2018) Connectome of the suprachiasmatic nucleus: new evidence of the core-shell relationship. eNeuro 5: ENEURO.0205-18.2018. 10.1523/ENEURO.0205-18.2018 PMC6168316 30283813

[bibr58-07487304221107834] Vasalou C Henson MA (2011) A multicellular model for differential regulation of circadian signals in the core and shell regions of the suprachiasmatic nucleus. J Theor Biol 288:44-56. 21871462 10.1016/j.jtbi.2011.08.010 PMC3184462

[bibr59-07487304221107834] Welsh DK Takahashi JS Kay SA (2010) Suprachiasmatic nucleus: cell autonomy and network properties. Ann Rev Physiol 72:551-577. 20148688 10.1146/annurev-physiol-021909-135919 PMC3758475

[bibr60-07487304221107834] Yamaguchi S Isejima H Matsuo T Okura R Yagita K Kobayashi M Okamura H (2003) Synchronization of cellular clocks in the suprachiasmatic nucleus. Science 302:1408-1412. 14631044 10.1126/science.1089287

[bibr61-07487304221107834] Yoon S Wright EAP Mendes JFF Goltsev AV (2021) Impact of field heterogeneity on the dynamics of the forced Kuramoto model. Phy Rev E 104:024313. 10.1103/PhysRevE.104.024313 34525638

[bibr62-07487304221107834] Yoshikawa T Pauls S Foley N Taub A LeSauter J Foley D Honma KI Honma S Silver R (2021) Phase gradients and anisotropy of the suprachiasmatic network: discovery of phaseoids. eNeuro 8: ENEURO.0078-21.2021. 10.1523/ENEURO.0078-21.2021 PMC8431825 34385151

